# Color-Vision and Color-Blindness

**Published:** 1896-05

**Authors:** 


Color-Vision and Color-Blindness. A Practical Manual for Railroad Surgeons. By J. Ellis Jennings, M. D. (University of Pennsylvania), formerly Clinical Assistant Royal London Ophthalmic Hospital (Moorfields) ; Lecturer on Ophthalmoscopy and Chief of the Eye Clinic in the Beaumont Hospital Medical College ; Ophthalmic and Aural Surgeon to the St. Louis Mullanphy and Methodist Deaconess Hospitals ; Consulting Oculist to the Missouri, Kansas & Texas Railway System ; Fellow of the British Laryngological and Rhinological Association. Illustrated with one colored full-page plate and twenty-one photo-engravings. Cloth, $1.00 net. Philadelphia : The F. A. Davis Co., Publishers. 1896.

This little work of 100 pages presents in an entertaining way the various color tests. The chapters are short and no space is taken up with the customary persiflage so rampant in the average medical article. The historical sketch, physiological anatomy of the retina, classification of color-blindness and the various tests are all very satisfactory. As he also takes up acuteness, range and field of vision with tests for hearing, it would seem to be a mistake to not also include marked muscular defects and the diagnosis of astigmatism, and thus make it what the work purports to be—"a complete manual for railway surgeons." R. H. S.

